# Nicotine exacerbates atherosclerosis and plaque instability via NLRP3 inflammasome activation in vascular smooth muscle cells

**DOI:** 10.7150/thno.81388

**Published:** 2023-05-08

**Authors:** Junqing An, Liu Ouyang, Changjiang Yu, Sean Michael Carr, Tharmarajan Ramprasath, Zhixue Liu, Ping Song, Ming-Hui Zou, Ye Ding

**Affiliations:** Center for Molecular and Translational Medicine, Georgia State University, 157 Decatur Street SE, Atlanta, GA 30303, USA.

**Keywords:** Nicotine, lysosomal dysfunction, NLRP3 inflammasome, atherosclerosis, unstable plaque, VSMC

## Abstract

**Rationale:** Nicotine has been reported to be a strong risk factor for atherosclerosis. However, the underlying mechanism by which nicotine controls atherosclerotic plaque stability remain largely unknown.

**Objective:** The aim of this study was to evaluate the impact of lysosomal dysfunction mediated NLRP3 inflammasome activation in vascular smooth muscle cell (VSMC) on atherosclerotic plaque formation and stability in advanced atherosclerosis at the brachiocephalic arteries (BA).

**Methods and Results:** Features of atherosclerotic plaque stability and the markers for NLR Family Pyrin Domain Containing 3 (NLRP3) inflammasome were monitored in the BA from nicotine or vehicle-treated apolipoprotein E deficient (*Apoe^-/-^*) mice fed with Western-type diet (WD). Nicotine treatment for 6 weeks accelerated atherosclerotic plaque formation and enhanced the hallmarks of plaque instability in BA of *Apoe^-/-^* mice. Moreover, nicotine elevated interleukin 1 beta (IL-1β) in serum and aorta and was preferred to activate NLRP3 inflammasome in aortic vascular smooth muscle cells (VSMC). Importantly, pharmacological inhibition of Caspase1, a key downstream target of NLRP3 inflammasome complex, and genetic inactivation of NLRP3 significantly restrained nicotine-elevated IL-1β in serum and aorta, as well as nicotine-stimulated atherosclerotic plaque formation and plaque destabilization in BA. We further confirmed the role of VSMC-derived NLRP3 inflammasome in nicotine-induced plaque instability by using VSMC specific TXNIP (upstream regulator of NLRP3 inflammasome) deletion mice. Mechanistic study further showed that nicotine induced lysosomal dysfunction resulted in cathepsin B cytoplasmic release. Inhibition or knockdown of cathepsin B blocked nicotine-dependent inflammasome activation.

**Conclusions:** Nicotine promotes atherosclerotic plaque instability by lysosomal dysfunction-mediated NLRP3 inflammasome activation in vascular smooth muscle cells.

## Introduction

Atherosclerosis is a chronic disease of the arterial wall that is responsible for nearly 50% of all deaths in developed countries 1-4. Despite the expenditure of billions of dollars and decades of research, there are still fundamental gaps in our knowledge of the mechanisms of the atherosclerotic development, progression, and end-stage clinical events, including plaque rupture. Plaque vulnerability is responsible for the sudden and unpredictable onset of acute coronary syndromes and lead to myocardial infarction and stroke. Cigarette smoking, which is one of the major preventable cause of premature death in the United States 5, is a major independent risk factor for cardiovascular disease 6-8. Atherosclerosis could be accelerated by cigarette smoking in the coronary arteries, aorta, carotid and cerebral arteries 9, 10. Nicotine, one of the major components of cigarette, is the primary addictive agent in cigarettes 11. Epidemiological studies have reported that the dose of nicotine consumption is proportional to the risk of cardiovascular diseases (CVD) related to atherosclerosis 12. In addition, nicotine has been shown to attract inflammatory cells onto endothelium and induce endothelial dysfunction 13, 14, which is an early marker of atherosclerosis. As the major constituent of electronic cigarettes, which are becoming popular as an alternative to tobacco smoking, the effect of nicotine on atherosclerotic plaque instability in advanced lesions, which in humans contribute to late-stage clinical events, is still undefined.

Inflammation is a critical component of atherosclerosis. Interleukin 1 (IL-1) is a classic pro-inflammatory cytokine that induces the production of cytokines and chemokines in vascular cells 15. Increases in sputum and lavage fluid interleukin 1 beta (IL-1*β*) have been documented in smokers compared with nonsmokers 16. Many clinical and experimental studies have also reported that IL-1*β* plays a crucial role in the progression of atherosclerosis and identified IL-1*β* as a proatherogenic cytokine 17, 18. More importantly, recent clinical trial data 19, 20 also found that specific targeting of IL-1*β* using antibody-canakinumab reduced heart disease and stroke by 15% by reducing inflammation. All these findings highlight the importance of the key role of cytokine IL-1*β* in cardiovascular disease. The processing and release of IL-1*β* are regulated by a multiprotein complex known as the inflammasome 21. Recently, the NLR Family Pyrin Domain Containing 3 (NLRP3) inflammasome has gained attention as a major contributor to several important cardiometabolic diseases including atherosclerosis. NLRP3 inflammasome is activated by endogenous or exogenous damage-associated molecular patterns and is involved in the process of sterile inflammation. Nevertheless, NLRP3 inflammasome has been reported to be activated by cholesterol crystals which are required for atherogenesis 22, whether NLRP3 inflammasome activation contributes to nicotine-induced atherosclerotic plaque instability is still unclear.

Vascular smooth muscle cells (VSMCs) are one of the major cell types present at all three stages of atherosclerosis including the initial step, progression and end-stage of an atherosclerotic plaque 23. Research in recent years has focused on the roles of VSMC apoptosis and VSMCs derived collagen and Extracellular matrix (ECM) regulation on plaque rupture 24. VSMCs are the only cells within plaques capable of synthesizing collagen which plays an important structural role in stabilizing plaques 25. VSMC apoptosis has been reported to induce multiple features of vulnerability to rupture in plaques 26. In addition, sterile inflammation can also be triggered in the VSMCs. Interestingly, Xin Shi et.al, reported that components of the NLRP3 inflammasome signaling pathway were strongly expressed in unstable human carotid atherosclerotic plaques 27. However, the role of VSMC-derived inflammasome-mediated vascular inflammation has not been fully elucidated in the pathogenesis of atherosclerotic plaque instability.

NLRP3 inflammasome is a vital component of the innate immune system and can be activated by different stimulation including ATP, Toll-like receptor ligands, mitochondrial dysfunction, the production of reactive oxygen species, and ionic flux 28, 29. In addition, lysosomal dysfunction is one of the critical factors which can activate NLRP3 inflammasome 30. Lysosomes are the cell's degradation center and are primarily responsible for the degradation of extracellular particles from endocytosis and of intracellular components from autophagy 31-33. Lysosome has been associated with diseases such as lysosomal storage disorders, neurodegenerative disorders, and cardiovascular disease 34, 35. Importantly, lysosomal dysfunction plays an important role in atherogenesis 36-38. However, whether lysosome dysfunction plays a role in regulating atherosclerotic plaque instability remains unknown. Lysosomal membrane permeabilization (LMP) potentiated lysosomal damage results in the release of its contents (cathepsins and other hydrolases) into the cytoplasm, inducing unselective degradation of cellular components 39-41. LMP-induced release of lysosome contents to cytoplasm has been reported to trigger NLRP3 inflammasome and subsequent downstream inflammation 42-44. However, it is unknown whether nicotine induces lysosomal impairment, and if nicotine-mediated lysosomal dysfunction contributes to NLRP3 inflammasome activation in VSMCs. Whether this mechanism is unique to nicotine priming and activation of NLRP3 remains to be investigated.

By employing pharmacological (inflammasome inhibitors) and genetic approaches (*NLRP3^-/-^* mice and VSMC specific TXNIP deletion mice), we aimed to determine the effect and molecular mechanisms of inflammasome activation on atherosclerosis and atherosclerotic plaque stability. Our results indicate that nicotine-induces lysosomal dysfunction in VSMCs that promotes atherogenesis and features of atherosclerotic plaque instability via activating NLRP3 inflammasome in VSMCs.

## Material and Methods

### Materials and Reagents

Antibodies to human NLRP3 (ab109314), Cathepsin B(ab92955) and LAMP1 (ab25245) were purchased from Abcam Technology. Antibodies to β-actin (sc-10731), Caspase1 (sc-56036), Caspase1 p10 (sc-514), and normal rabbit IgG (sc-2027) were obtained from Santa Cruz Biotechnology. Anti-mouse NLRP3 (AG-20B-0014-C100) antibody was purchased from AdipoGen. Antibody to Caspase1 p20 (4199s), proIL-1β (12242s), mIL-1β (83186s), TXNIP (67280), ASC (67824), and ASC (13833) was from Cell Signaling Technology. Antibodies to mouse IL-1β (AF-401-NA), human IL-1β (AF-201-NA) and mouse IL-1β Quantikine ELISA Kit (MLB00C) were obtained from R&D. Antibody to smooth muscle alpha-actin (A5691) for Immunohistochemistry (IHC) staining was from Sigma Aldrich. Antibody to Ter119 (14-5921-82) for Immunohistochemistry (IHC) staining was from ThermoFisher. All antibodies were used in a 1:1,000 dilution for Western blotting and a 1:100 dilution for IHC. A goat anti-Rat IgG conjugated to Alexa 555 (A21434) was purchased from Molecular Probes (Life Technologies). Nicotine (n3876-25 mL), minocycline hydrochloride (1444004), CA-074 methyl (C5857), NH_4_Cl (254134), and Masson trichrome staining kit (HT15A-1KT) were obtained from Sigma Aldrich. Bafilomycin A1 (BML-CM110-0100) was from Enzo Life Sciences. Scr siRNA (sc-37007) and siRNAs targeting human NLRP3 (sc-45469), human ASC (sc-37281), human Cathepsin B (sc-29238) were from Santa Cruz. LysoTracker® Red DND-99 (L-7528) was purchased from Life technologies. Magic Red™ Cathepsin B Assay Kit (937) was from Immunochemistry technologies. BODIPY® FL Histamine (B22461) was from Thermofisher. Caspase1 colorimetric assay kit (K111-100) was from BioVision. The transfection reagents for siRNA (Lipofectamine RNAiMax, 13778150) and all primers were from Invitrogen.

**Animal diet, feeding schedule and preparation of tissues.** *Apoe^-/-^* mice were obtained from the Jackson Laboratory (Bar Harbor, Maine, USA). All experimental procedures involving animals were approved by the Institutional Animal Care and Use Committee at Georgia State University. 8-week old male *Apoe^-/-^* mice were fed a Western diet containing 21% milk fat and 0.15% cholesterol for 6 weeks to establish aortic lesions, then mice were infused with nicotine (5 mg/kg/day) or vehicle (0.9% sodium chloride) for another 6 weeks using Alzet osmotic pumps (Model 2006, DURECT Corporation, Cupertino, CA) as described previously 45. For Caspase1 inhibition experiment, after 6 weeks of Western diet feeding, the *Apoe^-/-^* mice were treated with or without minocycline hydrochloride (10mg/kg/2days) by intraperitoneal injection immediately after vehicle- or nicotine-infusion and for the following 6 weeks. Similarly, 8-week old male *Apoe^-/-^Nlrp3^-/-^* mice, *Apoe^-/-^Txnip^sm22α-/-^* mice and *Apoe^-/-^* littermate control mice were placed on western diet for the initial 6 weeks and were treated vehicle or nicotine for another 6 weeks. Mice were sacrificed and blood was collected. Mice were then perfused via the left ventricle with 5 ml PBS followed by 10 ml 4% paraformaldehyde. Heart and brachiocephalic arteries (BA) were carefully dissected and fixed overnight in 4% paraformaldehyde prior to embedding in optimum cutting temperature compound (OCT; BDH Laboratory Supplies).

**Morphometric and immunohistochemical analysis of aortic root and brachiocephalic arteries (BA).** To analyze the lesion area in the BA, the aorta was dissected and cleaned, then OCT-embedded BA were serially sectioned at 8 μm thickness from the distal end where it branches into the right subclavian and right carotid for 480 μm. For morphometric and immunohistochemical analysis, sections of each BA were stained at 80 μm intervals from 0 to 480 μm distal to the aortic arch. For example, 0, 80, 160, 240, 320, 400 μm, 6 different locations of the BA were used for each specific type of staining and analysis. For incidence evaluation, if we observed the phenomenon in any one of the six sections, it was counted as one. For other analyses, mean value of the six different sections was collected. H&E staining was performed for analysis of the incidence of intraplaque hemorrhage, buried fibrous cap, discontinuity in the fibrous cap and calculating necrotic core size. Oil red O staining was performed for the analysis of plaque size in BA. Masson trichrome staining was performed for analysis of collagen content and fibrous cap by measuring the blue staining area in the images. Immunohistochemical staining (IHC) for α-SMA was conducted with detection by Permanent Red. All other IHC staining was detected by DAB. Olympus fluorescence microscope was used for collecting the images. Plaque size, necrotic core size and fibrous cap area were quantified by ImageJ (NIH). Collagen content, areas of positive IHC staining and optical density of positive IF staining were analyzed using Image-Pro Plus 6.0 (Media Cybernetics), as described previously 26, 46. All the analyses were performed in a blinded fashion.

**Vulnerability-Index**. Vulnerability-Index (VI) was used to evaluate the degree of plaque instability and was calculated as previous described 47, 48. VI was calculated by analyzing unstable (U) and stable (S) features of the plaque and corrected by the incidence of lesion formation (*p_(i)_*, VI_C_). The formula for VI was expressed as VI_C_(i)=(U_(i)_/S_(i)_) *p_(i)_, where i represents each studies mouse. U includes the sum of necrotic core area (% of plaque) and CD68^+^ area (% of plaque). S includes the sum of α-SMA^+^ area (% of plaque) and collagen^+^ area (% of plaque).

**Measurement of serum IL-1β levels.** Serum IL-1β levels were measured using the enzyme-linked immunosorbent assay (ELISA)kit from R&D according to the manufacturer's instructions.

**Cell culture and treatment.** At 70-80% confluency, human aortic smooth muscle cell (hASMC) grown in M231 medium were treated with different agents, as indicated. The purity of VSMCs was confirmed through positive staining for α-SMA. In all experiments, cells were used between passages 3 and 10. For experiments involving nicotine treatment, cells were treated with 0.5 μM nicotine for 24 h unless otherwise stated.

**Transfection of siRNA hASMC.** Transient transfection of siRNA was carried out according to manufacturer's instruction. Briefly, the siRNAs were dissolved in siRNA buffer (20 mM KCl; 6 mM HEPES, pH 7.5; 0.2 mM MgCl_2_) to prepare a 10 µM stock solution. HASMC grown in 6 well plates were transfected with siRNA in transfection medium (Gibcol) containing liposomal transfection reagent (Lipofectamine RNAimax, Invitrogen). For each transfection, 100 µl of transfection medium containing 4 µl siRNA stock solution was gently mixed with 100 µl transfection medium containing 4 µl transfection reagent. After a 30 min incubation at room temperature, siRNA-lipid complexes were added to the cells in 1.0 ml transfection medium, and cells were incubated with this mixture for 6 h at 37°C. The transfection medium was then replaced with normal medium, and cells were cultured for 48 h.

**Western blot analysis**. Cell lysates were subjected to Western blot analysis. From the whole aortas collected, adventitia and endothelium were removed and homogenates of the media of the aortic walls were used for Western blot analysis. The protein content was measured by BCA protein assay reagent (Pierce, USA). 30 μg protein was loaded to SDS-PAGE and then transferred to membrane. The membrane was incubated with a 1:1000 dilution of primary antibody, followed by a 1:5000 dilution of horseradish peroxidase-conjugated secondary antibody. Protein bands were visualized by ECL (GE Healthcare).

**Lysosome staining.** After stimulated with nicotine, live hASMC were stained with 500 nM LysoTracker Red or 10 µM BODIPY® FL Histamine in tissue culture medium for 15-30 min at 37°C as indicated by the manufacturer's instructions. Then images were observed using a confocal microscope (LSM800, Carl Zeiss Microscopy Ltd). The quantification of lysosomes was calculated by the mean fluorescence intensity (MFIs) of LysoTracker and BODIPY. Specifically, 6 samples of each group were stained with LysoTracker and BODIPY, and random 10 fields (40x magnification) of each sample were selected to calculate the mean fluorescence intensity by using Image J software.

**Caspase1 activity assay.** After nicotine stimulation, hASMC were harvested and Caspase1 activity was detected with Caspase1 Colorimetric Assay Kit according to the manufacturer's instructions.

**PCR for mRNA expression.** Total RNA was isolated using kit from Qiagen (RNeasy Mini Kit) and reverse transcribed to cDNA using the ThermoScript RT-PCR system protocol (Invitrogen). The primers used for RT-PCR are listed in Table S1.

**Statistics.** Quantitative results are expressed as mean ± SEM. Chi-Square test was applied to comparisons of intraplaque hemorrhage, buried fibrous cap and discontinuity of the fibrous cap incidence. After confirming that all variables were normally distributed by the Kolmogorov-Smirnov test followed by Q-Q plots analysis, statistical differences were determined by Student's t-test for comparison between two groups and two-way ANOVA analysis followed by Bonferroni's multiple comparison test for comparison among multiple groups. P values of less than 0.05 were considered statistically significant.

## Results

### Nicotine treatment aggravates atherogenesis in brachiocephalic artery (BA)

**Figure 1:**
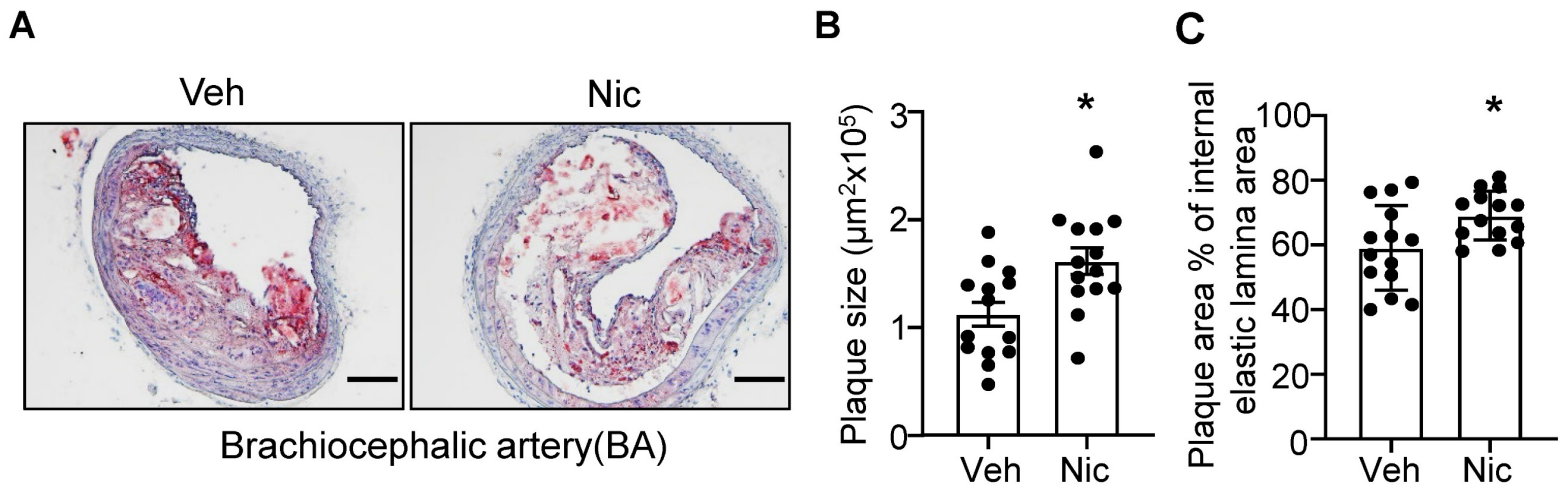
** Nicotine infusion increases atherosclerotic plaque sizes in BA.** (A) Oil red O staining of representative BA of *Apoe^-/-^* mice with vehicle or nicotine infusion. (B-C) Quantification of atherosclerotic plaque size (B) and plaque area percentage of internal elastic lamina area (C) in BA of *Apoe^-/-^* mice with vehicle or nicotine infusion. N=14 in each group. Scale bar: 100 μm. Values are represented as mean ± SEM *P<0.05 vs Veh. Veh, vehicle; Nic, nicotine.

To examine whether nicotine increases the atherogenesis in mice, we first analyzed the lesion size of the BA in atherosclerosis-susceptible *Apoe^-/-^* mice using Oil red O staining. As shown in **Figure 1A-C**, nicotine infusion for 6 weeks significantly increased the plaque size and plaque area percentage of internal elastic lamina area in BA compared with that of vehicle-treated mice. These data suggest that nicotine, the core component in cigarette smoking and electronic cigarette smoking, markedly aggravates atherogenesis in *Apoe^-/-^* mice.

### Nicotine stimulates features of atherosclerotic plaque instability

**Figure 2:**
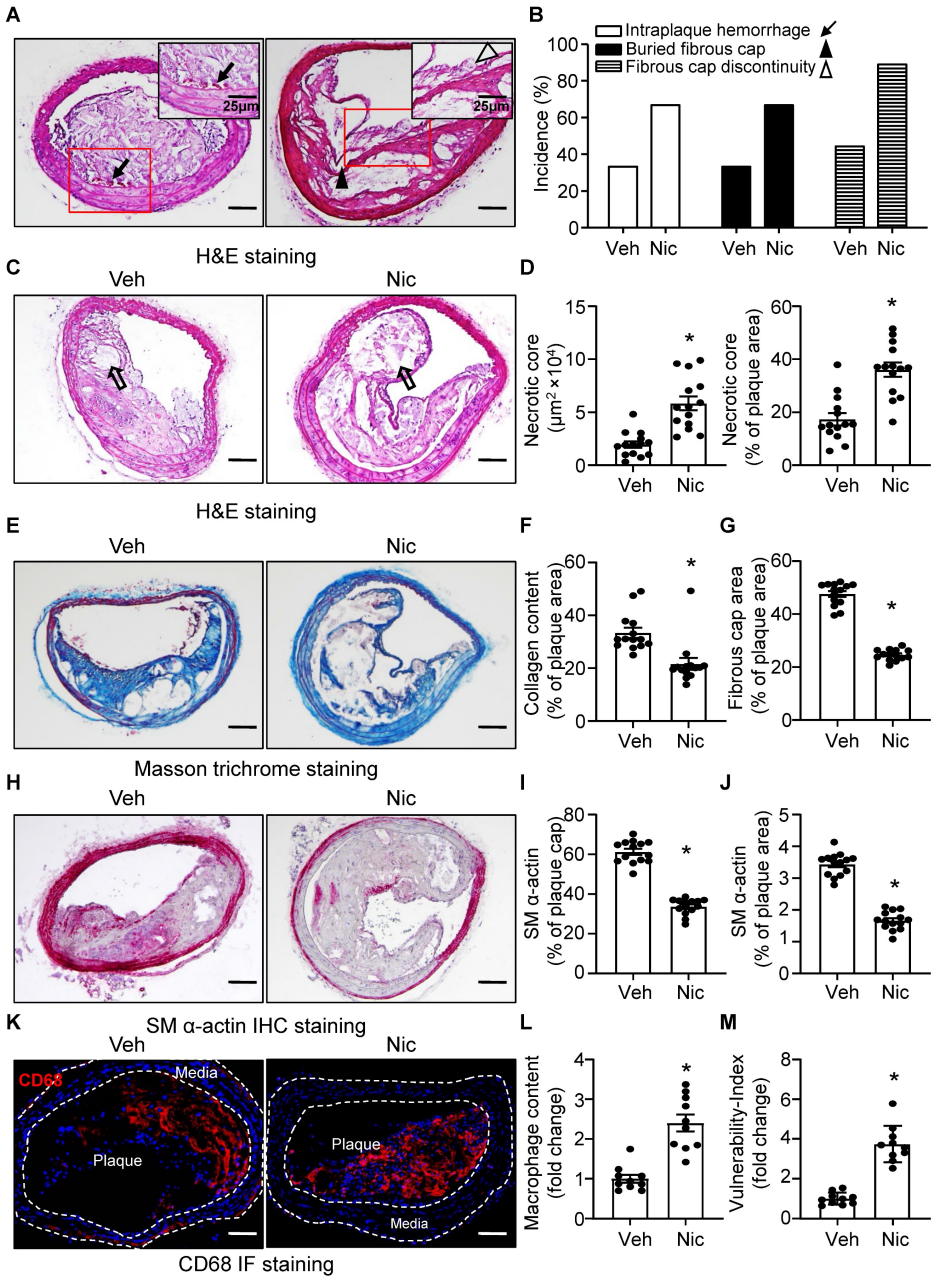
** Nicotine promotes features of atherosclerotic plaque vulnerability.** (A) Representative images from BA lesions of nicotine-infused mice with H&E staining for intraplaque hemorrhage (black arrow), buried fibrous cap (black arrowhead) and fibrous cap discontinuity (black hollow arrowhead). (B) Incidence for intraplaque hemorrhage, presence of buried fibrous cap and presence of fibrous cap discontinuity in the BA of *Apoe^-/-^* mice infused with vehicle or nicotine. N=18 in each group (C) Representative images of necrotic core area in the BA based on H&E staining (black hollow arrow) of *Apoe^-/-^* mice infused with vehicle or nicotine. (D) Quantification of necrotic core area in the BA of *Apoe^-/-^* mice infused with vehicle or nicotine. N=14. (E) Representative images of plaque collagen content in BA based on Masson trichrome staining (blue staining) of *Apoe^-/-^* mice infused with vehicle or nicotine. (F) Quantification of collagen content in the BA of *Apoe^-/-^* mice infused with vehicle or nicotine. N=14. (G) Quantification of the area of fibrous cap in BA of *Apoe^-/-^* mice infused with vehicle or nicotine. N=14. (H) Representative images of immunohistochemistry staining of SM α-actin (dark pink) in BA of *Apoe^-/-^* mice infused with vehicle or nicotine. (I) Quantification of plaque α-SMA (SM α-actin) coverage on the plaque cap in BA of *Apoe^-/-^* mice infused with vehicle or nicotine. N=14. (J) Quantification of total plaque SM α-actin content in BA of *Apoe^-/-^* mice infused with vehicle or nicotine. N=14. (K-L) Representative immunofluorescence staining and quantification of CD68 for inflammatory burden (macrophage content) in BA of *Apoe*^-/-^ mice infused with vehicle or nicotine. N=10. M, Quantification of vulnerability-index in BA of *Apoe*^-/-^ mice with vehicle or nicotine infusion. N=10. Scale bar: 100 μm. Values are represented as mean ± SEM. *P<0.05 vs Veh. Veh, vehicle; Nic, nicotine.

The phenotypic features of vulnerable plaques include increased intraplaque hemorrhage 49, 50, presence of buried fibrous cap 51, 52, discontinuity in the fibrous cap 53, enlarged lipid-rich necrotic core size, decreased thickness of fibrous cap 51 and plaque collagen content 54, 55, all of which have been widely used as indicators of plaque instability. To test whether nicotine influences the features of plaque stability, the aforementioned parameters were detected in the BA, a widely used artery for studying plaque stability or vulnerability in terms of an advanced atherosclerotic lesion in a mouse model 56. Intraplaque hemorrhage **(Figure 2A, black arrow)**, defined as the presence of erythrocytes (**Figure S1**) within the plaque, contributes to plaque instability as they promote oxidative stress and cholesterol accumulation 57, was significantly increased in nicotine-treated *Apoe^-/-^* mice relative to vehicle-treated *Apoe^-/-^* mice (**Figure 2A-B and Figure S1**); In addition, buried fibrous caps (**Figure 2A, black arrowhead**) that represents old plaque disruption that has healed 52, 58 dramatically increased in nicotine-treated mice compared with that of vehicle mice (**Figure 2A-B**). The existence of fibrous cap discontinuity (**Figure 2A, black hollow arrowhead**), which also called acute plaque rupture, defined as a visible breach in the cap 59, may directly reflect plaque rupture and was found increased in nicotine-treated *Apoe^-/-^* mice (**Figure 2A-B**). Furthermore, plaque necrosis which contributes to inflammation, thrombosis, physical stress on the fibrous cap, and plaque breakdown 60, were analyzed. The necrotic core (**Figure 2C, black hollow arrow**) size in nicotine-treated *Apoe^-/-^* mice was significantly increased relative to vehicle treatment (**Figure 2C-D**). Also, plaque collagen content, which plays an important structural role in stabilizing plaques 25, was decreased in nicotine-treated *Apoe^-/-^* mice relative to vehicle-treated mice (**Figure 2E-F**). Fibrous cap area, which is widely used as an indirect indicator of plaque stability, was markedly reduced in nicotine-treated *Apoe^-/-^* mice relative to vehicle-treated mice (**Figure 2G**), consistent with features of unstable plaques in humans 53. In addition, smooth muscle alpha-actin (SM α-actin) staining was dramatically weakened both in the plaque area and on the plaque cap in nicotine-treated mice than vehicle-treated mice (**Figure 2H-J**). The infiltration of macrophages also significantly increased in the plaque area in nicotine-treated mice (**Figure 2K-L**). Finally, we calculated the vulnerability-index that allowed us to integrate all parameters analyzed and determine the degree of vulnerability in vehicle and nicotine treated mice. We found nicotine-treated mice showed a significant higher vulnerability-index than that in vehicle-treated mice (**Figure 2M**). Taken together, all these results demonstrate that nicotine promotes features of an unstable plaque phenotype in advanced atherosclerosis.

### Nicotine triggers NLRP3 inflammasome activation in aorta and increases inflammatory cytokine IL-1β in serum

**Figure 3:**
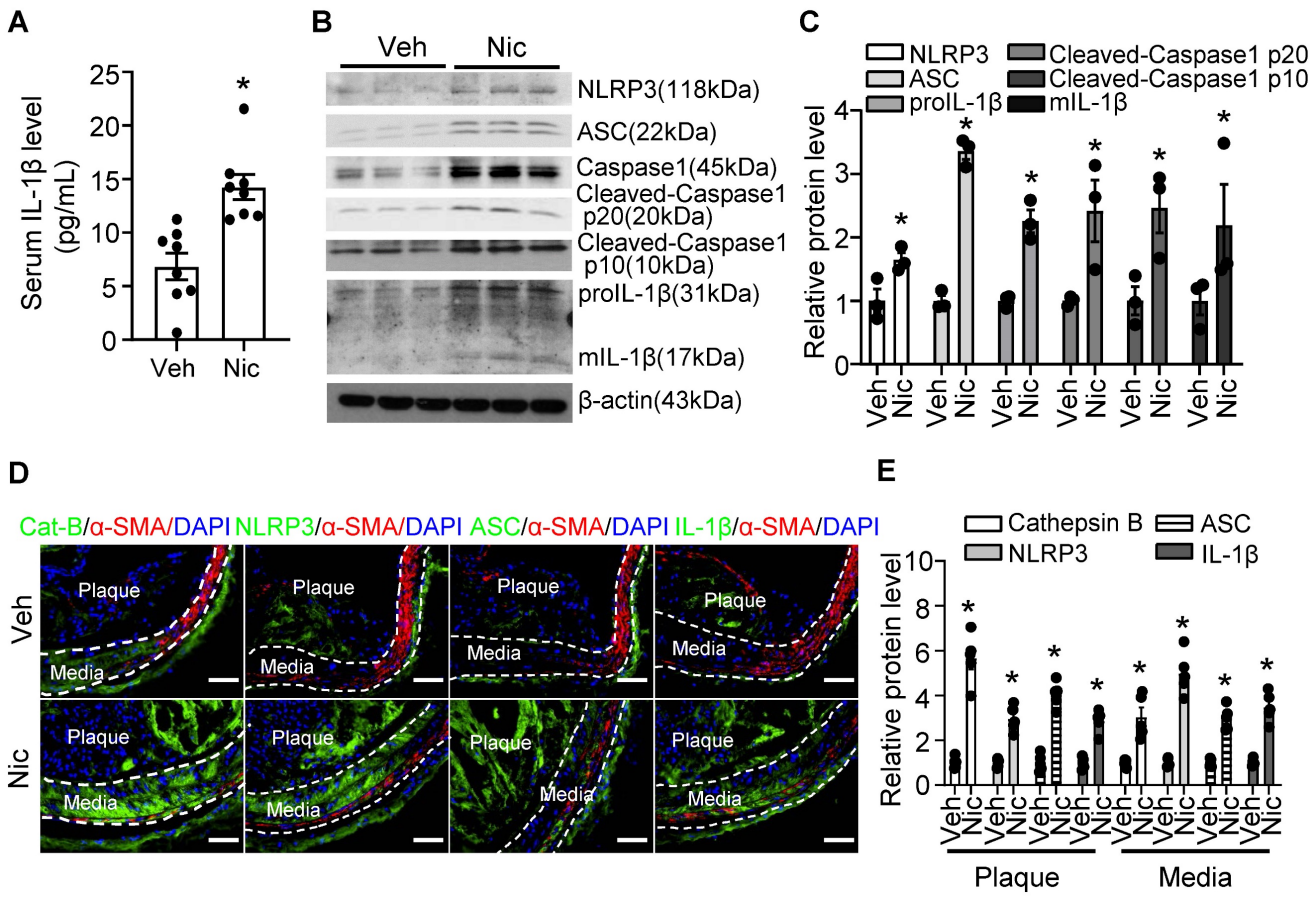
** Nicotine triggers NLRP3 inflammasome and increases inflammatory cytokine IL-1β in serum and aorta.** (A) ELISA detection of serum level of IL-1β in *Apoe^-/-^* mice with vehicle or nicotine infusion. N=8. (B-C) Western blot analysis and quantification of inflammasome markers in aorta from *Apoe^-/-^* mice with vehicle or nicotine infusion. N=3. (D) Immunofluorescence staining of co-localization between α-SMA and Cat-B (Cathepsin B), NLRP3, ASC, or IL-1β in BA of *Apoe^-/-^* mice with vehicle or nicotine infusion. (E) Quantification of relative Cathepsin B, NLRP3, ASC, and IL-1β expression in plaques and media of BA from Apoe-/- mice with vehicle or nicotine infusion. N=5. Scale bar: 50 μm. Values are represented as mean ± SEM. *P<0.05 vs Veh. Veh, vehicle; Nic, nicotine.

Vascular inflammation is considered as a key cause of atherogenesis. As shown in **Figure 3A**, serum levels of inflammatory cytokine IL-1β were markedly elevated in nicotine-infused mice. Furthermore, Western blotting data indicated that nicotine profoundly increased the protein expression of inflammasome components, NLRP3, ASC (apoptosis-assocaited speck-like protein containing CARD, also PYCARD), and Caspase1**,** as well as the expression of proIL-1β in aorta tissue (**Figure 3B-C**). The inflammasome activation also demonstrated by increased Caspase1 cleavage and matured IL-1β (mIL-1β) levels (**Figure 3B-C**). To further investigate the localization of the inflammasome component in the plaque, we performed immunofluorescence co-staining cell specific markers and inflammasome components in BA in vehicle and nicotine-treated mice. Consistently, nicotine enhanced the staining of anti-Cathepsin B, anti-ASC, NLRP3, and IL-1β in BA compared with that of vehicle-treated mice (**Figure 3D-E**). Interestingly, we observed that the positive signals of Cathepsin B, NLRP3, ASC and IL-1β were not only expressed in the plaque, but also expressed in the media of the BA, which are predominately composed of VSMCs (**Figure 3D**). However, the immunofluorescence co-localization analysis showed that nicotine had mild effects on inflammasome activation in endothelial cells, but not in the macrophages and T cells (**Figure S2**). Overall, the above results suggest that nicotine treatment are more likely to activate NLRP3 inflammasome in VSMCs in the atherosclerotic plaque in BA.

### Caspase1 inhibition partly blunts nicotine-induced atherogenesis and plaque instability

**Figure 4:**
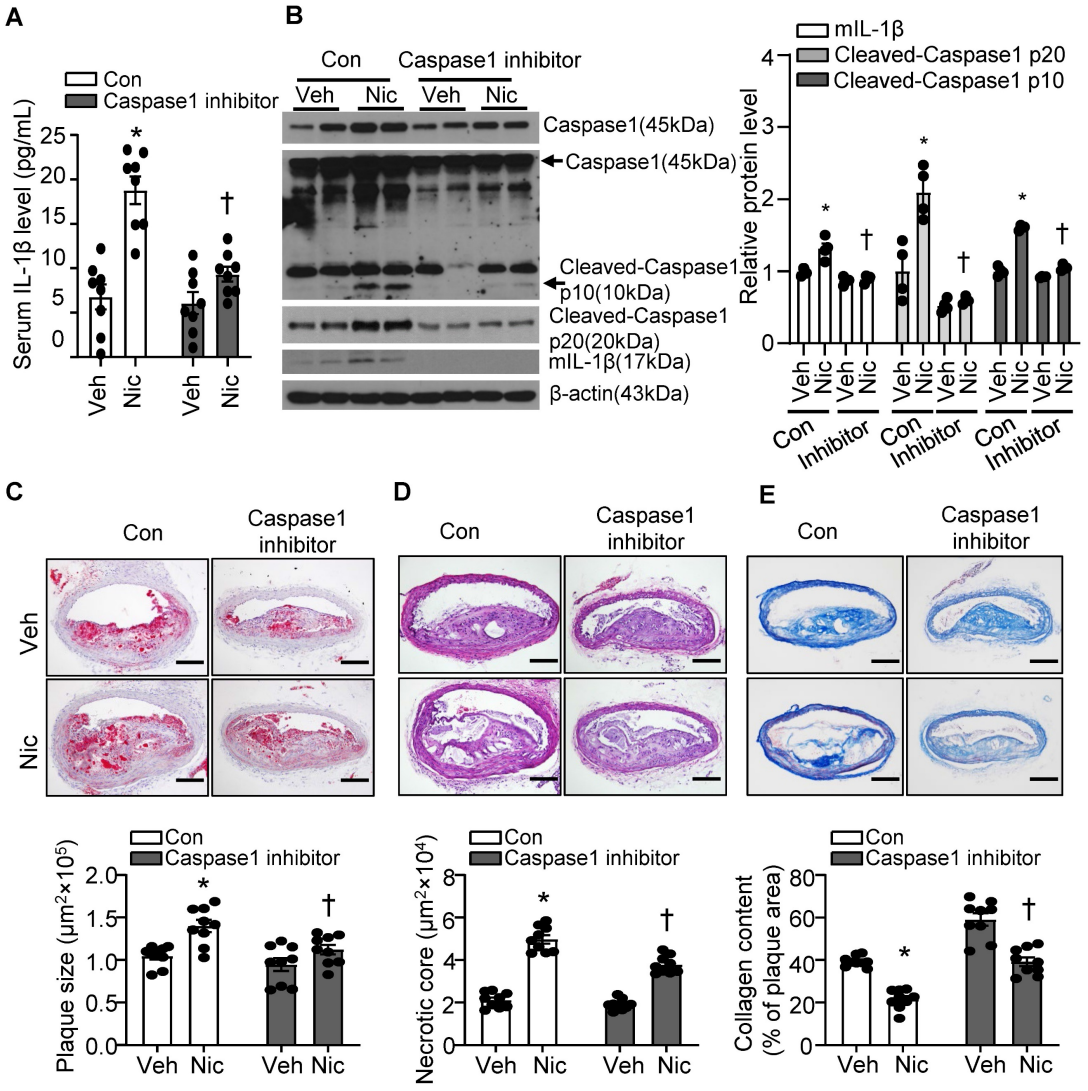
** Caspase1 inhibition partly blunts nicotine-induced atherogenesis and plaque vulnerability.** (A) Serum IL-1β level in vehicle- or nicotine-infused *Apoe^-/-^* mice treated with or without Caspase1 inhibitor. N=8. (B) Western blot analysis and quantification of inflammasome markers in aorta from vehicle- or nicotine-infused *Apoe^-/-^* mice treated with or without Caspase1 inhibitor. N=4. (C) (Top) Representative images from BA lesions with Oil Red O staining and (Bottom) quantification of plaque size of BA in vehicle- or nicotine-infused *Apoe^-/-^* mice treated with or without Caspase1 inhibitor. N=9. (D) (Top) Representative images and (Bottom) quantification of necrotic core area in the BA based on H&E staining of vehicle- or nicotine-infused *Apoe^-/-^* mice treated with or without Caspase1 inhibitor. N=9. (E) (Top) Representative images and (Bottom) quantification of plaque collagen content in BA based on Masson trichrome staining of vehicle- or nicotine-infused *Apoe^-/-^* mice treated with or without Caspase1 inhibitor. N=9. Scale bar: 100 μm. Values are represented as mean ± SEM. *P<0.05 vs. Veh. ^†^*P*<0.05 vs Nic+Con. Veh, vehicle; Nic, nicotine.

Caspase1 activation plays a pivotal role in inflammasome function. We treated mice with minocycline hydrochloride, a Caspase1 inhibitor 61, to test whether inflammasome signaling inhibition alleviates nicotine-mediated atherogenesis and atherosclerotic plaque instability. As shown in **Figure 4A**, Caspase1 inhibitor significantly decreased serum IL-1β levels elevated by nicotine, suggesting Caspase1 inhibitor restrains systemic inflammation induced by nicotine. In line with this finding, Caspase1 inhibitor not only clearly suppressed pro-Caspase1 expression in aorta, but also dramatically decreased Cleaved-Caspase1 p20/p10 and mIL-1β (**Figure 4B**). Next, we analyzed the effect of Caspase1 inhibitor on atherogenesis and atherosclerotic plaque instability. As shown in **Figure 4C-E**, Caspase1 inhibitor not only significantly reduced the atherosclerotic plaque size in *Apoe^-/-^* mice enhanced by nicotine (**Figure 4C**), but also markedly decreased nicotine-augmented necrotic core size (**Figure 4D**) and increased nicotine-reduced collagen content (**Figure 4E**) in *Apoe^-/-^* mice. All these data indicate that Caspase1 inhibition substantially ablates nicotine-induced atherosclerotic formation and plaque instability, further supporting that inflammasome activation plays an important role in nicotine-exacerbated atherogenesis and plaque destabilization.

### NLRP3 deletion alleviates nicotine-induced atherogenesis and plaque vulnerability

**Figure 5:**
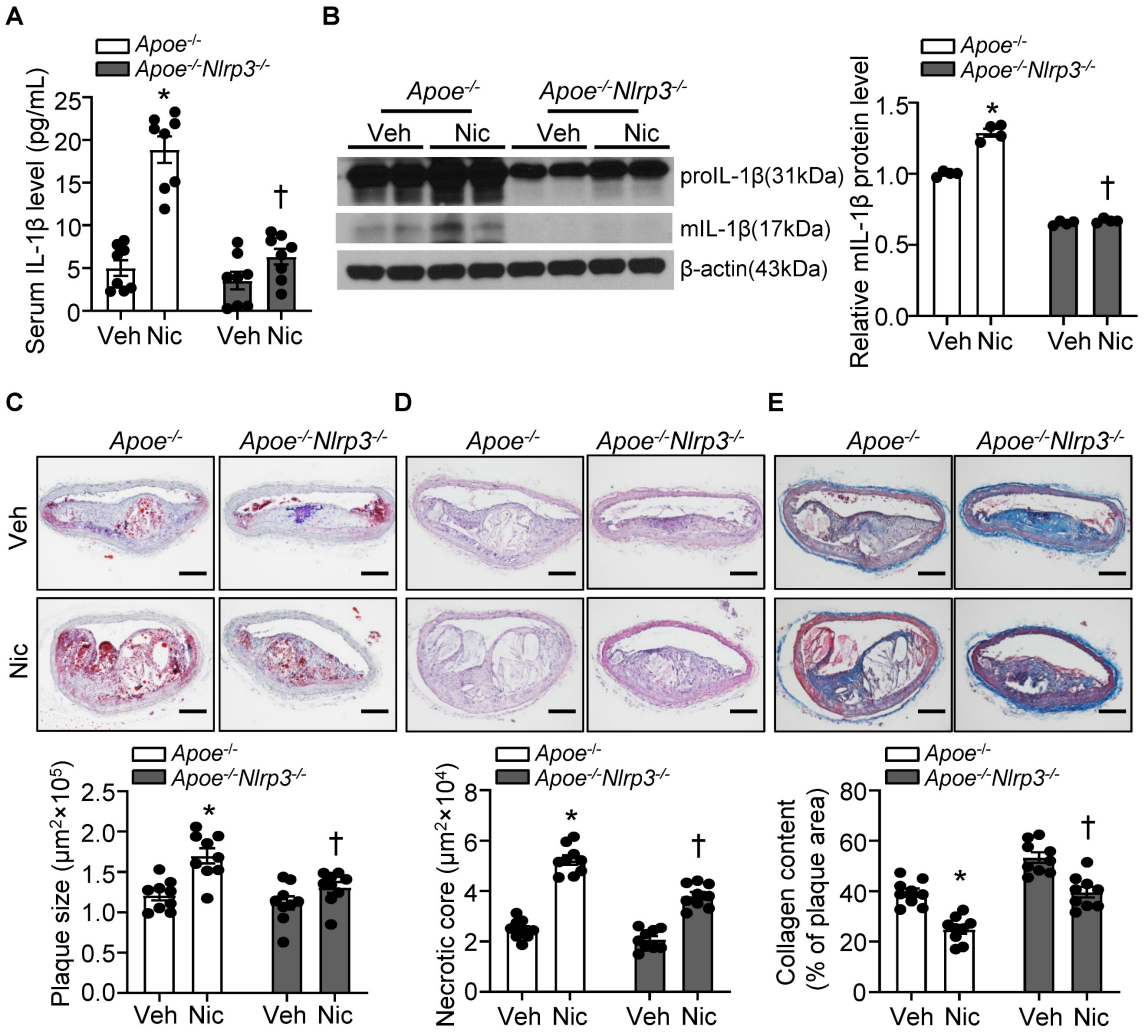
** NLRP3 deletion alleviates nicotine-induced atherogenesis and plaque vulnerability.** (A) Serum IL-1β level in vehicle- or nicotine-infused *Apoe^-/-^* mice or *Apoe^-/-^Nlrp3^-/-^* mice. N=8. (B) Western blot analysis and quantification of inflammasome markers in aorta from vehicle- or nicotine-infused *Apoe^-/-^* or *Apoe^-/-^ Nlrp3^-/-^* mice. N=4. (C) (Top) Representative images from BA lesions with Oil Red O staining and (Bottom) quantification of plaque size of BA in vehicle- or nicotine-infused *Apoe^-/-^* mice or *Apoe^-/-^ Nlrp3^-/-^* mice. N=9. (D) (Top) Representative images and (Bottom) quantification of necrotic core area in the BA based on H&E staining of vehicle- or nicotine-infused *Apoe^-/-^* mice or *Apoe^-/-^ Nlrp3^-/-^* mice. N=9. (E) (Top) Representative images and (Bottom) quantification of plaque collagen content in BA based on Masson trichrome staining of vehicle- or nicotine-infused *Apoe^-/-^* mice or *Apoe^-/-^ Nlrp3^-/-^* mice. N=9. Scale bar: 100μm. Values are represented as mean ± SEM. *P<0.05 vs. *Apoe^-/-^* mice Veh. ^†^*P*<0.05 vs* Apoe^-/-^* mice Nic. Veh, vehicle; Nic, nicotine.

To ascertain the role of NLRP3 inflammasome in nicotine-mediated plaque vulnerability, we generated mice which lack NLRP3 in *Apoe^-/-^* background. As shown in **Figure 5A-B**, *Apoe^-/-^Nlrp3^-/-^* mice exposed to nicotine displayed alleviated circulating IL-1β level as well as decreased mIL-1β expression in aorta compared with that in nicotine-exposed *Apoe^-/-^* control mice, indicating a positive association between NLRP3 inflammasome-mediated inflammation and nicotine-induced plaque instability. Further study demonstrated that NLRP3 deletion significantly lessened nicotine infusion-increased atherogenesis in BA (**Figure 5C**). In addition, *Apoe^-/-^Nlrp3^-/-^* mice exposed to nicotine showed markedly decreased necrotic core size (**Figure 5D**) and increased collagen content (**Figure 5E**) in BA compared with that in nicotine-exposed *Apoe^-/-^* control mice. These data demonstrated that NLRP3 inflammasome activation was required for the release of mIL-1β in atherosclerotic plaque in BA and contributed to nicotine-promoted atherogenesis and plaque instability.

### Thioredoxin-interacting protein (TXNIP) deletion in VSMC alleviates nicotine-induced atherogenesis and plaque vulnerability

**Figure 6:**
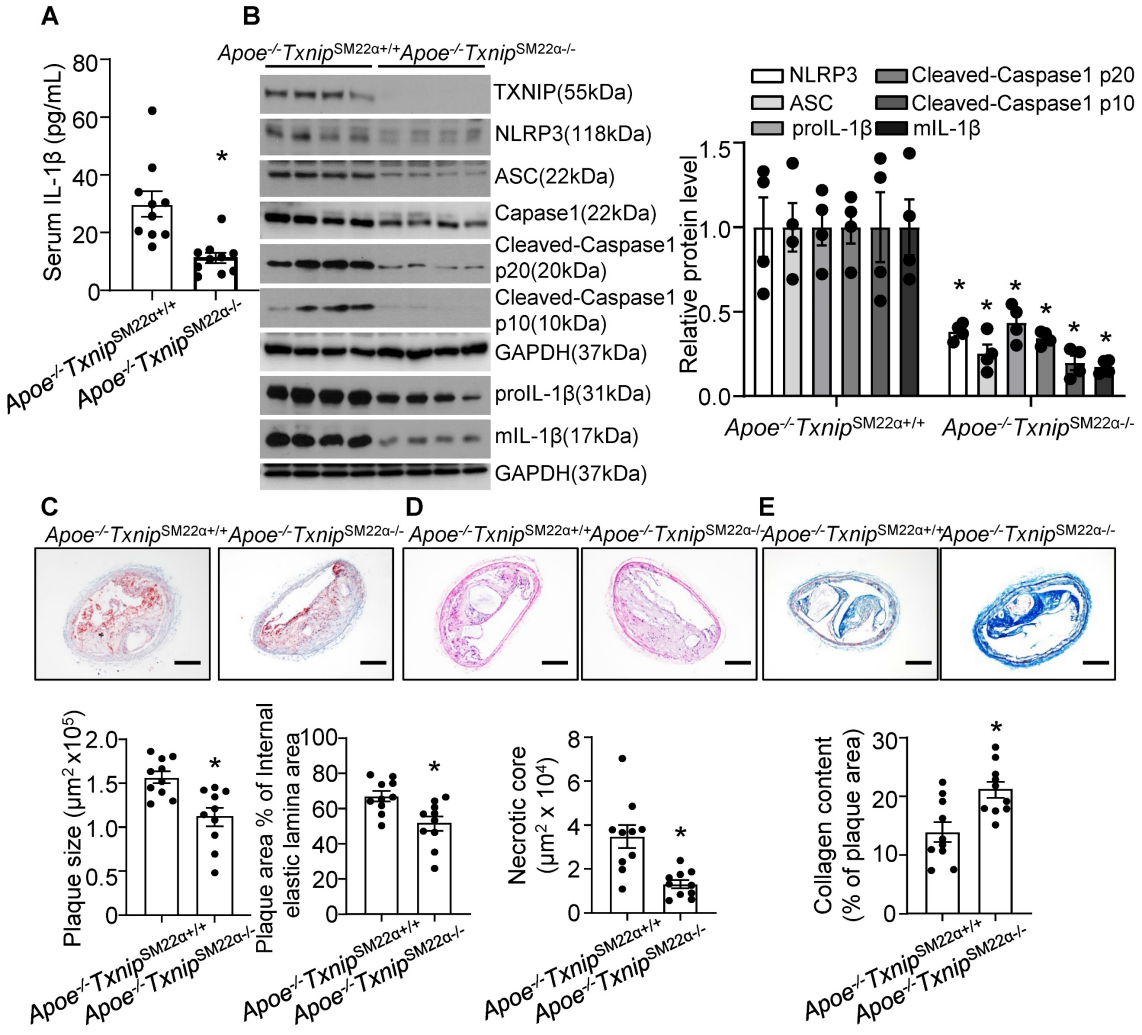
** TXNIP deletion in smooth muscle cells alleviates nicotine-induced atherogenesis and plaque vulnerability.** (A) Serum IL-1β level in nicotine-infused *Apoe^-/-^Txnip^SM22α+/+^* mice and *Apoe^-/-^Txnip^SM22α-/-^* mice. N=10. (B) Western blot analysis and quantification of NLRP3, ASC, Cleaved-Caspase1 p10, Cleaved-Caspase1 p20, pro-IL-1β and mIL-1β in aorta from nicotine-infused *Apoe^-/-^Txnip^SM22α+/+^* mice and *Apoe^-/-^Txnip^SM22α-/-^* mice. N=4. (C) (Top) Representative images from BA lesions with oil Red O staining and (Bottom) quantification of plaque size and plaque area percentage of internal elastic lamina area of BA in nicotine-infused *Apoe^-/-^Txnip^SM22α+/+^* mice and *Apoe^-/-^Txnip^SM22α-/-^* mice. N=10. (D) (Top) Representative images and (Bottom) quantification of necrotic core area in the BA based on H&E staining of nicotine-infused *Apoe^-/-^Txnip^SM22α+/+^* mice and *Apoe^-/-^Txnip^SM22α-/-^* mice. N=10. (E) (Top) Representative images and (Bottom) quantification of plaque collagen content in the BA based on Masson trichrome staining of nicotine-infused *Apoe^-/-^Txnip^SM22α+/+^* mice and *Apoe^-/-^Txnip^SM22α-/-^* mice. N=10. Scale bar:100μm. Values are represented as mean ± SEM. *P<0.05 vs.* Apoe^-/-^Txnip^SM22α+/+^* mice.

VSMC is one of the major cell type which participates in atherogenesis and plaque stability 23, 62. TXNIP is an upstream partner to NLRP3 and the interaction between TXNIP and NLRP3 was necessary for NLRP3 inflammasome activation 63. Next, to evaluate the contribution of VSMC-derived NLRP3 inflammasome in nicotine-induced atherosclerotic plaque vulnerability, we generated VSMC-specific TXNIP deletion mice in Apoe^-/-^ background. We first evaluated IL-1β level in serum and aorta. As shown in **Figure 6A-B**, *Apoe^-/-^Txnip^SM22α-/-^* mice exposed to nicotine displayed alleviated circulating IL-1β level as well as decreased inflammasome components NLRP3, ASC, Caspase1, Cleaved-Caspase1 p10, Cleaved-Caspase1 p20, proIL-1β and mIL-1β expression in aorta compared with that in nicotine exposed *Apoe^-/-^ Txnip^SM22α+/+^* control mice, suggesting VSMC-derived NLRP3 inflammasome-mediated inflammation might be responsible for nicotine-induced plaque instability. Then we evaluated the effect of TXNIP in VSMC on atherogenesis in the BA. Oil-red-O staining results showed that atherosclerotic plaque size within the BA of *Apoe^-/-^Txnip^SM22α-/-^* mice exposed to nicotine was decreased compared with those of *Apoe^-/-^ Txnip^SM22α+/+^* mice (**Figure 6C**). As depicted in **Figure 6D**, *Apoe^-/-^Txnip^SM22α-/-^* mice exposed to nicotine displayed significantly decreased necrotic core size compared with that of nicotine-exposed* Apoe^-/-^ Txnip^SM22α+/+^* mice. In parallel, increased collagen content was evident in *Apoe^-/-^Txnip^SM22α-/-^* mice relative to *Apoe^-/-^ Txnip^SM22α+/+^* mice exposed to nicotine (**Figure 6E**). Moreover, *Apoe^-/-^Txnip^SM22α-/-^* mice exposed to nicotine displayed improved α-SMA (SM α-actin) expression in the plaque area compared to *Apoe^-/-^ Txnip^SM22α+/+^* mice (**Figure S3**). These results demonstrate that *Apoe^-/-^Txnip^SM22α-/-^* mice displayed alleviated features of plaque instability similar to those in the BA of global *Apoe^-/-^Nlrp3^-/-^* mice under nicotine exposure condition. These results strongly suggest that VSMC-derived NLRP3 inflammasome is required for nicotine-enhanced atherogenesis and plaque destabilization.

### Nicotine activates the NLRP3 inflammasome and elevates inflammatory cytokine IL-1β in human VSMCs

**Figure 7:**
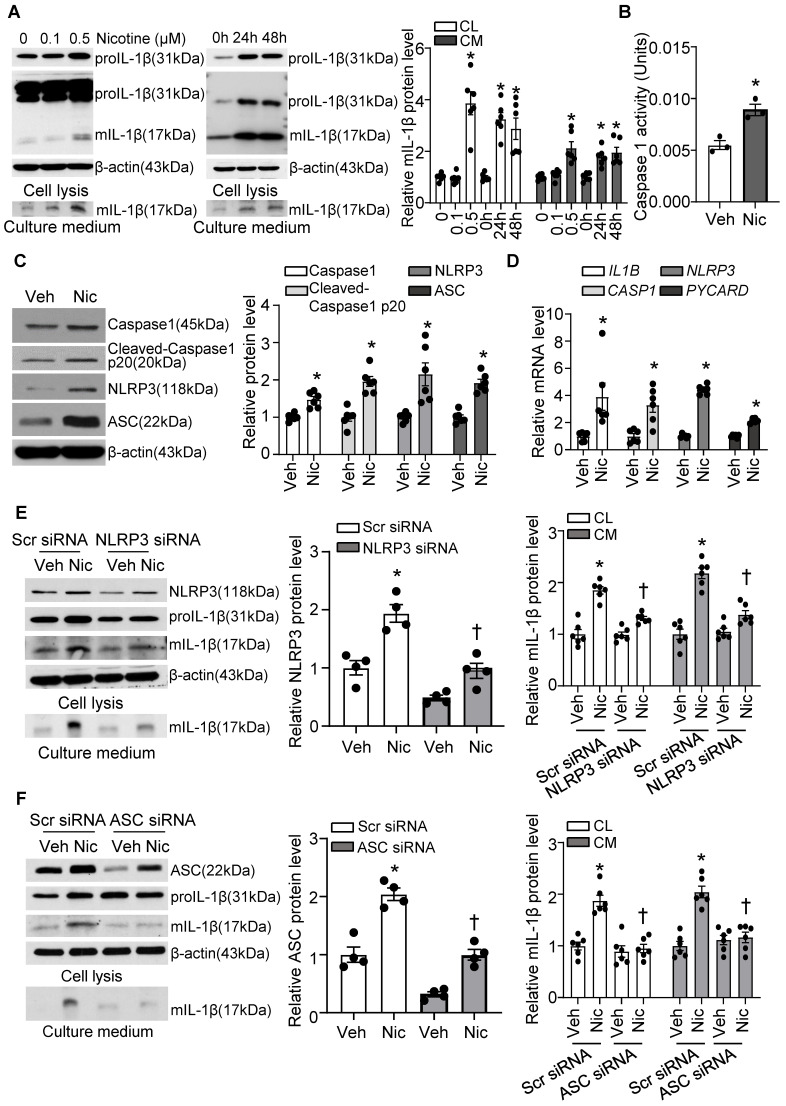
** Nicotine primes and activates NLRP3 inflammasome in human VSMC.** (A) Nicotine increased mIL-1β in both cell lysis (CL) and cell culture medium (CM) of hASMCs. N=6. (B) Nicotine increased Caspase1 activity in hASMC (nicotine, 0.5 μM for 24 h). N=3 (C) Western blot analysis and quantification of inflammasome markers in vehicle and nicotine-treated hASMC (nicotine, 0.5 μM for 24 h). N=6. (D) Quantitative real-time PCR of inflammasome markers in vehicle and nicotine-treated hASMC (nicotine, 0.5 μM for 24 h). N=6. (E) NLRP3 siRNA inhibited mIL-1β in cell lysis and culture medium induced by nicotine (nicotine, 0.5 μM for 24 h). N=6. (F) ASC siRNA blocked mIL-1β secretion in cell lysis and culture medium induced by nicotine (nicotine, 0.5 μM for 24 h). N=6. CL: Cell lysis; CM: Culture medium. Veh, vehicle; Nic, nicotine. Values are represented as mean ± SEM. *P<0.05 vs. 0, Veh or Veh-Scr siRNA. ^†^*P*<0.05 vs Nic-Scr siRNA.

Next, we investigated if nicotine triggers NLRP3 inflammasome activation in hASMCs. As shown in **Figure 7A**, nicotine profoundly elevated proIL-1β and mIL-1β in cell lysis, as well as mIL-1β in culture medium under nicotine treatment. IL-1β maturation and secretion are mediated by inflammasome dependent activation of Caspase1. Consistently, we found nicotine significantly increased Caspase1 activity in hASMC as shown in **Figure 7B**. Moreover, nicotine treatment impressively increased the protein expression of inflammasome components NLRP3, ASC, and Caspase1, as well as cleaved Caspase 1 p20 (**Figure 7C**), suggesting nicotine triggers NLRP3 inflammasome activation in hASMC. In addition, nicotine also notably elevated the mRNA levels of *NLRP3*, *PYCARD*, *CASP1*, and *IL-1B* (**Figure 7D**). Taken together, these data indicate that nicotine plays a critical role in NLRP3 inflammasome activation in hASMC involves both priming and activating steps.

To further confirm the involvement of NLRP3 inflammasome in nicotine-induced mIL-1β secretion, we silenced NLRP3 and ASC respectively in hASMC. As expected, siRNA-mediated NLRP3 knockdown substantially normalized nicotine elevated mIL-1β in VSMCs lysis and culture medium (**Figure 7E**). Similarly, ASC knockdown also remarkably normalized nicotine-elevated mIL-1β secretion in VSMCs lysis and culture medium (**Figure 7F**). These data suggest that NLRP3 inflammasome activation is responsible for nicotine-induced mIL-1β secretion in hASMC.

As macrophage and endothelial cells also exert their important roles in atherogenesis and plaque vulnerability, we tested the effect of nicotine on mIL-1β production in macrophage and endothelial cells. Firstly, we treated murine macrophages (RAW 264.7) and HUVECs (human umbilical vein endothelial cells) with 0.5 μM nicotine (same dose as VSMCs) and used LPS as the positive control. We found nicotine showed very mild effects on the activation of proIL-1β and mIL-1β in macrophages and HUVECs (**Figure S4A-B**). To further confirm the role of nicotine in human monocytes and endothelial cells, human monocytes (THP-1) and HAoECs (human aortic endothelial cells) were treated with same dose of nicotine. We found nicotine treatment increased IL-1β production in HAoECs but not in THP-1 cells (**Figure S5A-B**). However, the increased fold of mIL-1β in HAoECs is much less than that in hASMCs (**Figure S5C**). Taken together, all these results suggest that VSMCs are more sensitive to nicotine for NLRP3 inflammasome activation than endothelial cells and macrophages.

### Nicotine induced lysosomal membrane permeabilization (LMP) and caused lysosome dysfunction in VSMCs

**Figure 8:**
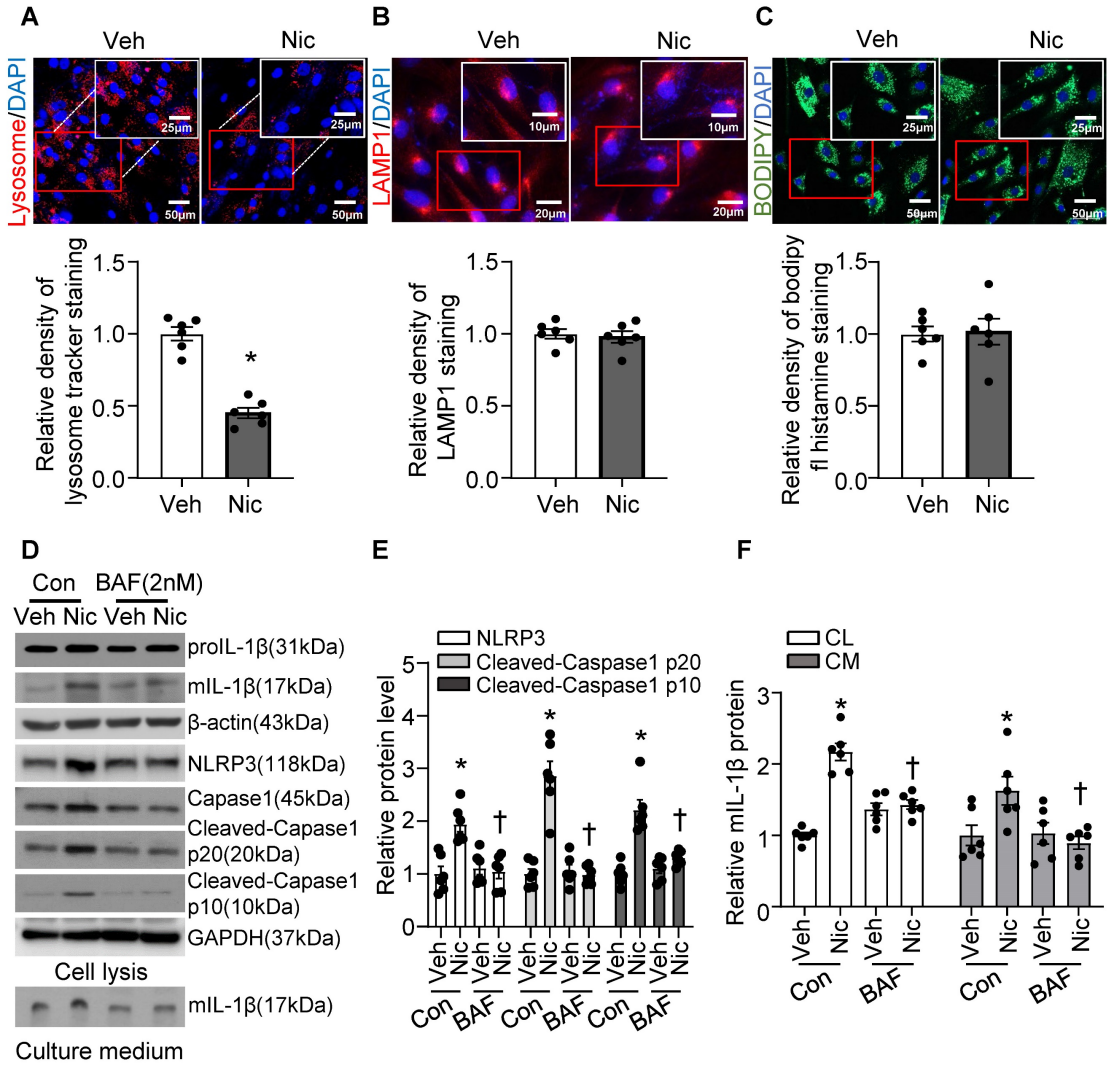
** Nicotine-induced lysosome dysfunction and elevated IL-1β resease in hASMC.** (A) Immunofluorescence images by LysoTrackerTM red in hASMC treated with or without nicotine (nicotine, 0.5 μM for 24 h). N=6. (B) Immunofluorescence images of LAMP1 in hASMC treated with or without nicotine (nicotine, 0.5 μM for 24 h). N=6. (C) Immunofluorescence images by PH-insensitive Tracker in hASMC treated with or without nicotine (nicotine, 0.5 μM for 24 h). N=6. (D-F) hASMCs were treated with vehicle or nicotine together with or without bafilomycin (BAF, 2 nM). Western blot analysis and quantification of NLRP3, Cleaved-Caspase1 p10, and Cleaved-Caspase1 p20 in cell lysis, and mature IL-1β in both cell lysis and culture medium. (nicotine, 0.5 μM for 24 h). N=6. CL: Cell lysis; CM: Culture medium. Veh, vehicle; Nic, nicotine. Values are represented as mean ± SEM. *P<0.05 vs. Veh or Veh-Con. ^†^*P*<0.05 vs Nic-Con.

Recent studies indicate that lysosome destabilization and subsequent leakage of lysosomal contents into the cytosol play a central role in NLRP3 activation 28, 64. To ascertain the role of lysosomal dysfunction in nicotine-mediated NLRP3 inflammasome activation in VSMCs, we first investigated the effect of nicotine on lysosome function. As shown in **Figure 8A**, nicotine treatment significantly decreased the staining density of LysoTracker Red, implying that either a loss of lysosomal acidity by nicotine leads to poor retention of the dye or a disruption in membrane integrity results in the lysosomal leakage or loss of lysosome. However, nicotine did not change the protein levels of lysosome-associated membrane glycoprotein 1 (LAMP1), a well-known lysosome marker (**Figure 8B**). Data staining with lysosome pH in-sensitive marker indicated that nicotine did not alter lysosome mass in hASMC (**Figure 8C**). These results suggested that nicotine might lead to lysosomal membrane permeabilization (LMP) which impairs lysosome function but not lysosome biogenesis. Low dose bafilomycin A (BAF), a potential LMP inhibitor 65, suppressed nicotine-induced NLRP3-inflammasome activation as well as IL-1β maturation and release in hASMC (**Figure 8D-F**). Furthermore, pharmacologically altering lysosome pH by NH_4_Cl treatment increased the basal and nicotine-mediated IL-1β secretion in hASMC (**Figure S6**). All of these data strongly suggested that lysosome dysfunction is required for nicotine-induced NLRP3 inflammasome activation in hASMC.

### Cathepsin B release is required for nicotine-induced NLRP3 inflammasome activation

**Figure 9:**
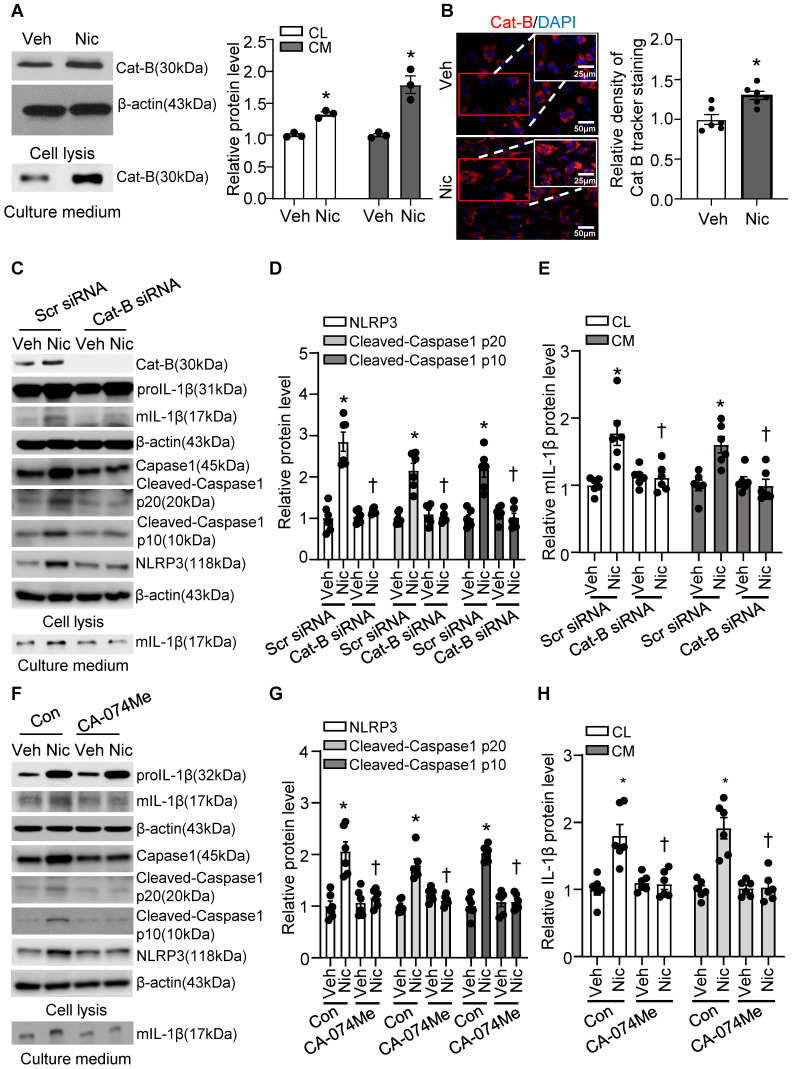
** Nicotine promoted Cathepsin B release and Cathepsin B inhibition suppressed nicotine induced NLRP3 inflammasome activation.** (A) Nicotine promoted Cathepsin B production and release to cell culture (nicotine, 0.5 μM for 24 h). N=3. (B) Immunofluorescence staining showing nicotine increased Cathepsin B activity in hASMCs (nicotine, 0.5 μM for 24 h). N=6. (C-E) hASMCs were treated with vehicle or nicotine together with control siRNA or cathepsin B siRNA. Western blot analysis and quantification of NLRP3, Cleaved-Caspase1 p10, and Cleaved-Caspase1 p20 in cell lysis, and mature IL-1β in both cell lysis and culture medium. (nicotine, 0.5 μM for 24 h). N=6. (F-H) hASMCs were treated with vehicle or nicotine together with or without cathepsin B inhibitor CA-074Me. Western blot analysis and quantification of NLRP3, Cleaved-Caspase1 p10, and Cleaved-Caspase1 p20 in cell lysis, and mature IL-1β in both cell lysis and culture medium. (nicotine, 0.5 μM for 24 h, CA-074Me, 3.5 nM for 24 h). N=6. Cat-B, Cathepsin B. CL: Cell lysis; CM: Culture medium. Veh, vehicle; Nic, nicotine. Values are represented as mean ± SEM. *P<0.05 vs. Veh or Veh-Scr siRNA or Veh-Con. ^†^*P*<0.05 vs Nic-Scr siRNA or Nic-Con.

Cathepsin B is a lysosomal protease that has been implicated in inflammasome activation following treatment with lysosome-damaging compounds such as silica and cholesterol crystals 66. As shown in **Figure 9A-B**, exogenous nicotine not only dramatically upregulated Cathepsin B in both cell lysis and culture medium, but also significantly increased Cathepsin B activity in hASMC, implying that Cathepsin B may be the contributor to nicotine-induced NLRP3 inflammasome activation.

To further address whether Cathepsin B release contributes to nicotine-induced NLRP3 inflammasome activation, we were genetically knockdown Cathepsin B with siRNA transfection and pharmacologically blocked Cathepsin B with specific inhibitor CA-074 Me in hASMC before treatment with nicotine, and then detected NLRP3-inflammasome activation and mIL-1β secretion 67. As shown in **Figure 9C-E**, Cathepsin B siRNA markedly decreased nicotine-elevated NLRP3-inflammasome activation as well as mIL-1β in both cell lysis and cell culture medium compared with control siRNA. Furthermore, Cathepsin B inhibitor CA-074 Me strikingly decreased nicotine-upregulated NLRP3-inflammasome activation as well as mIL-1β in both cell lysis and culture medium (**Figure 9F-H**). All these results imply that Cathepsin B mediates NLRP3 inflammasome activation in response to nicotine in hASMC.

## Discussion

The clinically important consequence of coronary atherosclerosis is the rupture or disruption of a “vulnerable” plaque 68. The current study demonstrates for the first time that nicotine promotes the features of atherosclerotic plaque instability in mice *in vivo*. We showed that nicotine impairs lysosome function, and the dysfunctional lysosome activates the NLRP3 inflammasome via Cathepsin B in VSMCs. Furthermore, NLRP3 inflammasome inhibition by Caspase1 inhibitor or NLRP3 deletion ameliorates nicotine-deteriorated atherogenesis and plaque destabilization. Most importantly, nicotine exposed *Apoe^-/-^Txnip^SM22α-/-^* mice exhibited alleviative atherogenesis and features of plaque instability compared to nicotine exposed *Apoe^-/-^Txnip^SM22α+/+^* mice. Taken together, our results strongly support that lysosomal dysfunction induced by nicotine contributes to NLRP3 inflammasome activation in VSMCs which consequently accelerated features of atherosclerotic plaque instability.

Vulnerable plaques are characterized by infiltrated inflammatory leukocytes in combination with a large necrotic core covered with a thin fibrous cap 69. Even though multiple atherosclerotic mouse models have been established to investigate the features of human advanced lesions 48, a consensus model of atherosclerotic plaque destabilization is still lacking. The hyperlipidemic mouse models we used here resemble some features of advanced human plaques including a large necrotic core, reduced collagen content, and accumulated macrophage infiltration. This model is extensively used in studying the mechanisms of the initiation and progression of atherosclerosis. However, they are lacking some of the vulnerable plaque features, such as plaque rapture and thrombotic events 48, 70. Additionally, the BA has been reported as the only site that could display multiple features of plaque instability in high-fat diet-fed mice 59. Hence, we focused on BA for studying plaque vulnerability in terms of an advanced atherosclerotic lesion as a useful mouse model.

There has been substantial interest in identifying pathways that lead to plaque destabilization and in the development of novel therapies that stabilize the vulnerable plaque and reduce acute coronary events. Atherosclerotic lesions in proximal aortas and aortic tree are profoundly decreased in IL-1 receptor 1 knockout with ApoE heterozygote (*Apoe^+/-^/IL-1R1^-/-^*) mice than in *Apoe^+/-^/IL-1R1^+/-^* mice challenged with *Porphyromonas gingivalis*, an important periodontal pathogen, or high-fat diet (HFD) 71. These results indicate that proinflammatory cytokine IL-1 plays a causal role in bacteria or HFD stimulated atherogenesis. However, advanced atherosclerotic plaques in mice lacking both IL-1 receptor type I and apolipoprotein E unexpectedly exhibits multiple features of plaque instability as compared with those of control mice 46. These studies suggest that IL-1 signaling plays a surprising dual role in mediating atherogenesis and atherosclerotic plaque vulnerability 46. In our present study, we found that nicotine exposure dramatically elevated IL-1β level in serum, in aorta, in plaque, and in the media layer of BA. Moreover, nicotine treatment significantly increased mIL-1β release in VSMCs *in vitro*. Importantly, VSMC specific deletion of TXNIP significantly reduced nicotine-induced IL-1β level both in serum and in the aorta. Our findings indicate that VSMCs not only participate in the process of atherogenesis and plaque stability as the major components of the vessel media, but also serves as the initiator of the sterile inflammatory response in atherosclerosis-related immune responses. And the detrimental role of VSMC-derived IL-1 signaling in atherosclerotic plaque instability also suggests IL-1β as a predictive biomarker and a potential therapeutic target to treat smoking patients with unstable atherosclerotic plaques.

Another major finding of this study is we demonstrate that NLRP3 inflammasome-derived IL-1β contributes to nicotine-mediated atherosclerotic plaque instability. Inflammasomes, which are a group of cytosolic protein complexes, are key signaling platforms that detect pathogenic microorganisms and sterile stressors to activate the highly pro-inflammatory cytokines IL-1β 72. Among those inflammasomes, NLRP3 inflammasome has been well characterized and its activation strongly links to sterile inflammation in a variety of chronic degenerative diseases including atherosclerosis 73. Dr. Wu. *et al* has reported that nicotine activated NLRP3 inflammasome and induced pyroptosis of endothelial cells which enhanced the atherosclerosis 74. However, whether NLRP3 inflammasome activation-mediated inflammation plays a causal role in nicotine-induced plaque instability is still unclear. Dr. Lau *et.al* has demonstrated that nicotine promotes atherogenesis by triggering proinflammatory responses in macrophages 75. They found significantly enhanced serum inflammatory cytokines TNFα and IL-1β in nicotine-exposed mice. However, in their *in vitro* studies, they detected no difference of IL-1β in both peritoneal macrophages isolated from nicotine-exposed mice and nicotine-treated Raw264.6 macrophages, which is consistent with our findings, suggesting exogenous nicotine could not directly trigger inflammasome activation in macrophages. Here in our present study, we observed significantly increased protein expression of NLRP3, ASC and IL-1β in both the plaque area and in media (which are majorly VSMCs) of the BA in nicotine exposed mice, which established the co-relation between NLRP3 inflammasome activation in VSMCs and unstable plaque. Furthermore, the *in-vivo* co-localization analysis and *in-vitro* experiments indicated that VSMCs were more sensitive to nicotine-induced NLRP3 inflammasome activation macrophages, T cells and endothelial cells. Intriguingly, selective inhibition of NLRP3 inflammasome activation in VSMCs both *in vitro* and *in vivo* by genetic or pharmacological strategies blocked the nicotine-induced mIL-1β elevation. Those data indicate that VSMC-derived NLRP3 inflammasome activation does play an important role in promoting atherogenesis and atherosclerotic plaque instability induced by nicotine. Even though, employment of the cell type-specific NLRP3-knockout transgenic models would precisely identify the target cell type that contributes to the nicotine-induced plaque instability. Taken together, this finding provides a new mechanistic insight into nicotine-induced atherosclerotic plaque instability and helps to identify VSMCs derived-NLRP3 inflammasome-IL1β as a potential therapeutic target to treat smoking patients with unstable atherosclerotic plaques.

Accumulating studies have reported that lysosomal function is markedly impaired in atherosclerosis, and that trigger of lysosomal dysfunction contributes to the pathogenesis of atherosclerosis 36-38. Lysosome rupture is associated with the release of proteases into the cytoplasm and, has been reported as an upstream signal for NLRP3 inflammasome activation 64. Recently, increasing evidence demonstrates that Cathepsin B (a major protease in lysosome) serves to amplify the in-progress inflammatory response in macrophages in atherosclerotic plaque 22. However, whether lysosome dysfunction plays a role in regulating atherosclerotic plaque instability is still unknown. Until now, most of the studies were focused on the contribution of macrophage-derived lysosome dysfunction to atherogenesis. It is unclear whether lysosome in VSMCs (the major cell type which participates in all three stages of atherosclerosis) plays critical roles in mediating atherogenesis and atherosclerotic plaque instability. It is also unknown whether nicotine induces lysosome impairment and nicotine-mediated lysosomal dysfunction in VSMCs contributes to atherogenesis and atherosclerotic plaque instability. In our present study, we observed that nicotine caused LMP which impaired lysosome function and lead to Cat B release to the cytoplasm. In addition, we found lysosomal inhibition or Cathepsin B specific inhibition strikingly decreased nicotine-upregulated mIL-1β secretion in hASMC. We report for the first time that LMP-Cathepsin B in VSMCs might be responsible for nicotine-triggered NLRP3 inflammasome activation and nicotine-enhanced atherosclerotic plaque instability, which provides potential therapeutic targets in preventing atherosclerotic plaque instability in smoking patients.

In conclusion, the results of the present study provide evidence that nicotine promotes atherogenesis and atherosclerotic plaque instability via lysosome dysfunction mediated-NLRP3 inflammasome activation in VSMCs. The findings of this study will help identify inhibition of lysosome dysfunction as a novel target and offer an innovative therapeutic strategy to prevent atherosclerosis initiation, progression, and rupture in smoking and secondhand smoking patients.

## Supplementary Material

Supplementary figures and table.
