# Supplementary material for: Nicotine exacerbates atherosclerosis and plaque instability via NLRP3 inflammasome activation in vascular smooth muscle cells
Source: Theranostics. 2023 May 8;13(9):2825–42. doi: 10.7150/thno.81388 (PMC10240824; doi:10.7150/thno.81388)
Supplement: Supplementary file 1 — Supplementary figures and table. [file thnov13p2825s1.pdf]

SUPPLEMENTAL MATERIAL

Nicotine exacerbates atherosclerosis and plaque instability via NLRP3 inflammasome activation in vascular smooth muscle cells

Junqing An[#], Liu Ouyang[#], Changjiang Yu, Sean Michael Carr, Tharmarajan Ramprasath, Zhixue Liu, Ping Song, Ming-Hui Zou, Ye Ding*

Center for Molecular and Translational Medicine, Georgia State University,157 Decatur Street SE, Atlanta, GA 30303

Supplemental Figures

Supplemental Figure 1

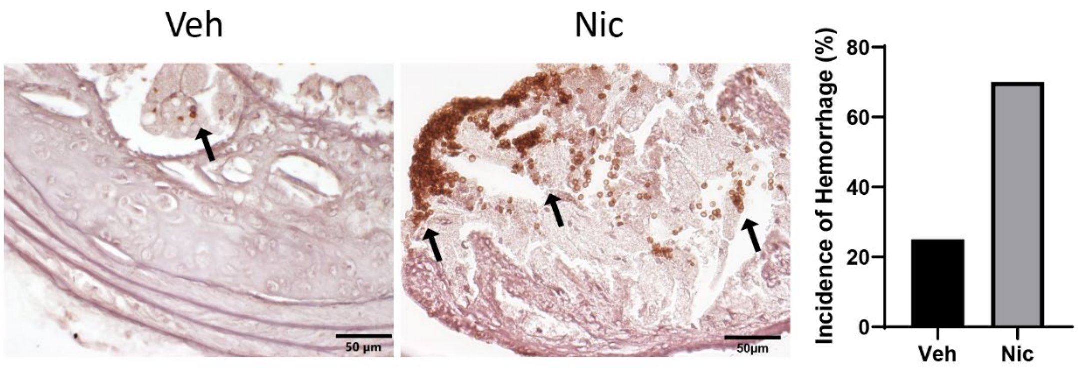

Figure S1. Red blood cells in atherosclerotic plaques. Representative immunohistochemistry staining of Ter119 for red blood cells and quantification of hemorrhage incidence in BA of *Apoe*$^{-/-}$ mice with vehicle or nicotine infusion. Scale bar: 50 µm. Veh, vehicle; Nic, nicotine.

Supplemental Figure 2

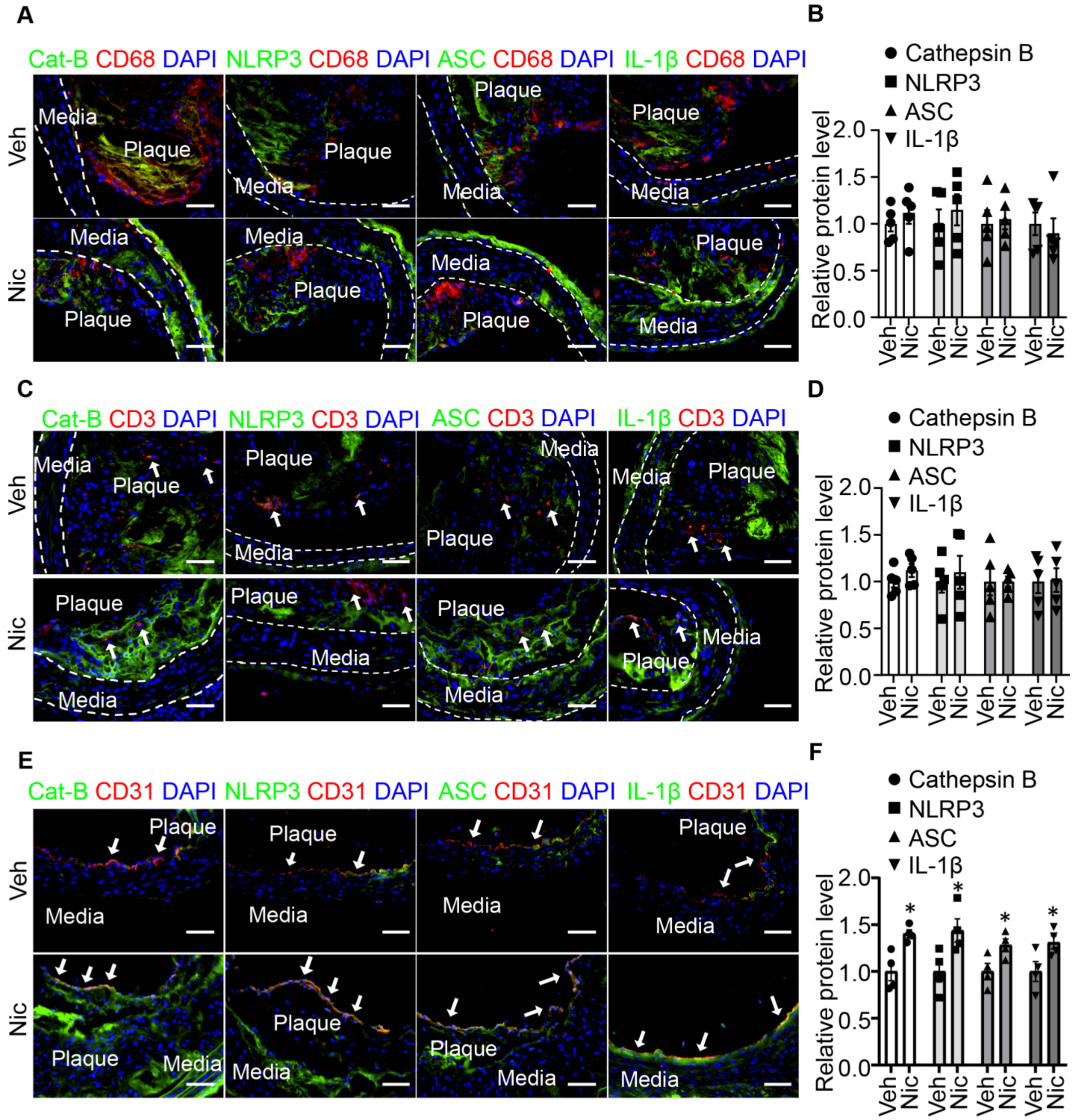

Figure S2. The effect of nicotine on inflammasome activation in macrophages, T cells and endothelial cells. (A-B), Immunofluorescence staining and quantification of CD68 and Cat-B, NLRP3, ASC, or IL-1β in BA of *Apoe*$^{-/-}$ mice with vehicle or nicotine infusion. N=5. Scale bar: 50 μm. (C&D), Immunofluorescence staining and quantification of CD3 and Cat-B, NLRP3, ASC, or IL-1β in BA of *Apoe*$^{-/-}$ mice with vehicle or nicotine infusion. N=5. Scale bar: 50 μm. (E-F),

Immunofluorescence staining and quantification of CD31 and Cat-B, NLRP3, ASC, or IL-1β in BA of *Apoe*$^{-/-}$ mice with vehicle or nicotine infusion. N=4. Scale bar: 50 μm. Cat B: Cathepsin B. Veh, vehicle; Nic, nicotine. Values represent the mean ± SEM. *P<0.05 vs Veh.

Supplemental Figure 3

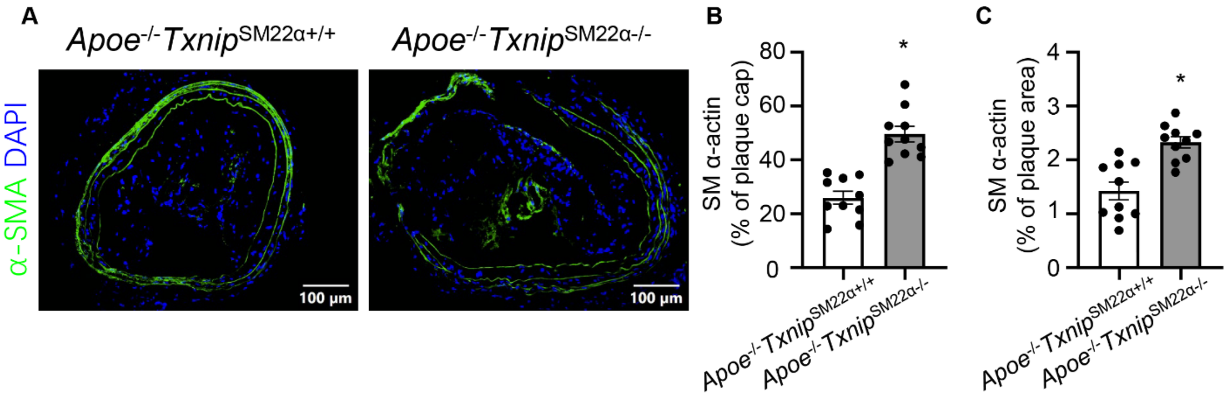

Figure S3. TXNIP deletion in smooth muscle cells improves α-SMA expression in nicotine-induced atherogenesis and plaque vulnerability. (A) Representative images of immunofluorescence staining of α-SMA (SM α-actin) in BA of *Apoe*$^{-/-}$*Txnip*$^{SM22\alpha+/+}$ and *Apoe*$^{-/-}$*Txnip*$^{SM22\alpha-/-}$ mice infused with nicotine. (B) Quantification of plaque SM α-actin coverage on the plaque cap in BA of *Apoe*$^{-/-}$*Txnip*$^{SM22\alpha+/+}$ and *Apoe*$^{-/-}$*Txnip*$^{SM22\alpha-/-}$ mice infused with nicotine. (C) Quantification of total plaque SM α-actin content in BA of *Apoe*$^{-/-}$*Txnip*$^{SM22\alpha+/+}$ and *Apoe*$^{-/-}$*Txnip*$^{SM22\alpha-/-}$ mice infused with nicotine. N=10. Scale bar: 100 μm. Values represent the mean ± SEM. *P<0.05 vs *Apoe*$^{-/-}$*Txnip*$^{SM22\alpha+/+}$. Veh, vehicle; Nic, nicotine.

Supplemental Figure 4

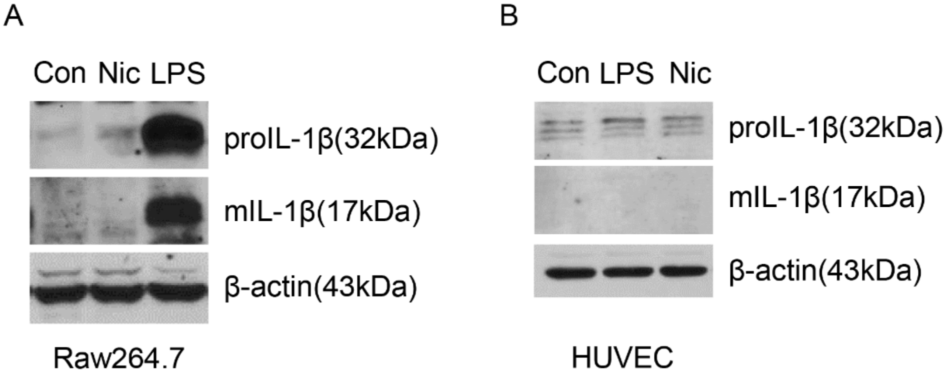

Figure S4. Nicotine did not increase inflammatory cytokine IL-1β in macrophage and HUVEC. (A-B) Western blot analysis of IL-1β in nicotine (0.5 μM) treated macrophage Raw264.7 (A) and HUVECs (B) for 24 hours. 100 ng/mL LPS served as control. Nic, nicotine.

Supplemental Figure 5

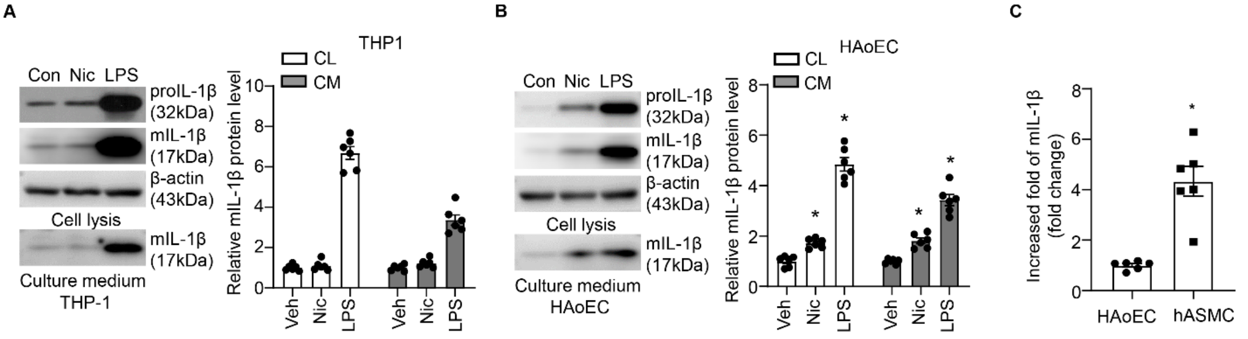

Figure S5. The effects of nicotine on inflammatory cytokine IL-1β in THP-1 and HAEC. (A) Western blot analysis and quantification of IL-1β release in nicotine (0.5 μM for 24 h) treated THP-1 cells. N=3. (B) Western blot analysis and quantification of IL-1β release in nicotine (0.5 μM for 24 h) treated human aortic endothelial cells (HAoECs). N=6. (C) Relative increased fold of mIL-1β in HAoECs and VSMCs. N=6. 100 ng/mL LPS for 24 hours served as control. Values represent the mean ± SEM. *P<0.05 vs Veh. Veh, vehicle; Nic, nicotine.

Supplemental Figure 6

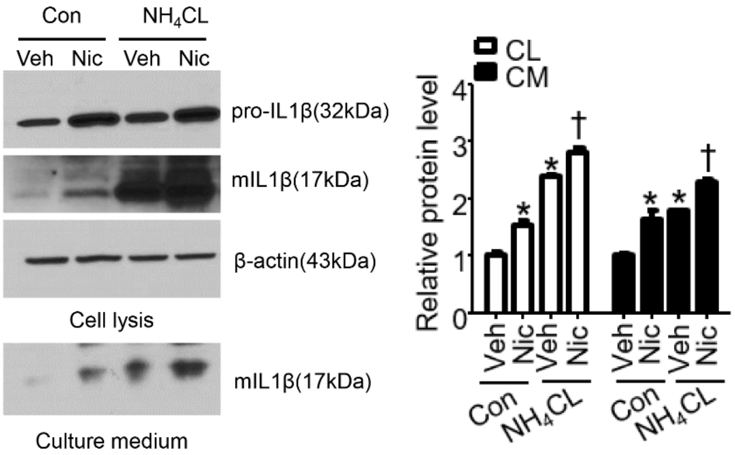

Figure S6. Lysosomal acidification inhibitor NH_4Cl further increased mIL-1β secretion both in cell lysis and cell culture medium induced by nicotine. VSMCs were treated with nicotine (0.5 μM for 24 h) together with or without NH_4Cl (20 mM for 24 h). Western blot analysis of IL-1β. Values represent the mean ± SEM. **P*<0.05 vs. Veh-Con. †*P*<0.05 vs Nic-Con. Veh, vehicle; Nic, nicotine.

Supplemental Table 1. Primer sequences used in real-time quantitative PCR.

Sequence no.	Gene name	Primer sequence
1.	Human 18S	F-5'-GTAACCCGTTGAACCCCATT-3'<br>R-5'-CCATCCAATCGGTAGTAGCG-3'
2.	Human NLRP3	F-5'-CTTCTCTGATGAGGCCCAAG-3'<br>R-5'-GCAGCAAACTGGAAAGGAAG-3'
3.	Human ASC	F-5'-CTCTGTACGGGAAGGTCCTG-3'<br>R-5'-TCCTCCACCAGGTAGGACTG-3'
4.	Human Caspase1	F-5'-TTCTGCTCTTCCACACC-3'<br>R-5'-CTACCATCTGGCTGCTC-3'
5.	Human IL-1β	F-5'-AAGGGCTGCTTCCAAACCT-3'<br>R-5'-ATACTGCCTGCCTGAAGCT-3'
